# Supplementary material for: Invariance of object detection in untrained deep neural networks
Source: Front Comput Neurosci. 2022 Nov 3;16:1030707. doi: 10.3389/fncom.2022.1030707 (PMC9669311; doi:10.3389/fncom.2022.1030707)
Supplement: Supplementary file 1 [file Data_Sheet_1.pdf]

Supplementary Material

1 Supplementary Figures and Tables

1.1 Supplementary Figures

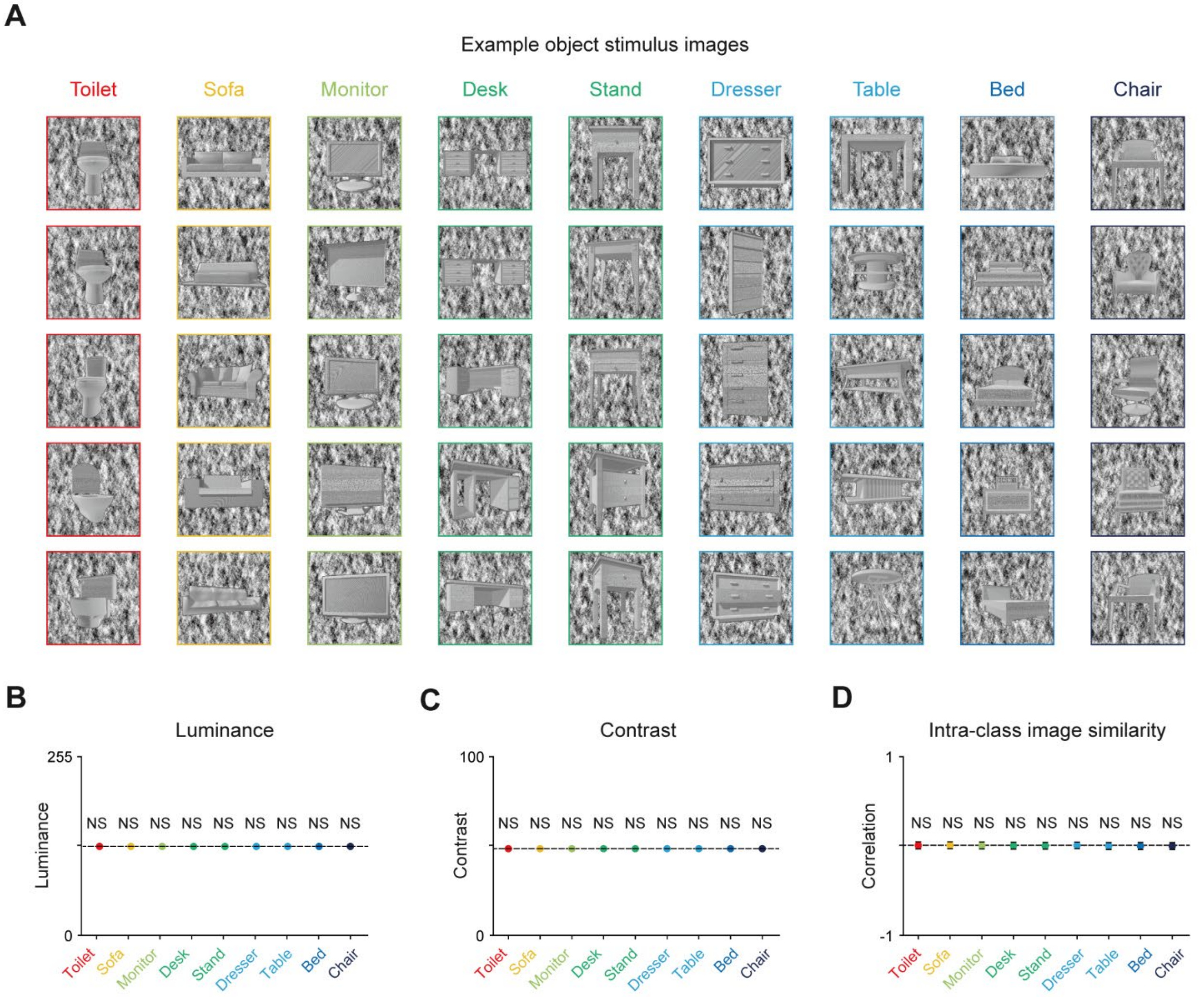

Supplementary Figure 1. Low-level feature-controlled object image stimulus: **(A)** Example images of the object dataset. An object stimulus renderer was used to create object images with different viewpoints. Object stimulus images were selected and adapted from a publicly available dataset that contains nine object classes. Each object set contains 200 different object identities rendered with viewpoint variations of ±30°. The original CAD models are available at https://modelnet.cs.princeton.edu/. **(B)** Controlled luminance of stimulus images (n = 200, two-sided rank-sum test, NS, P > 0.829). The luminance of an image was measured as the mean of the pixel intensity. **(C)** Controlled contrast of object stimulus (n = 200, two-sided rank-sum test, NS, P > 0.507). The contrast of an image was measured as the standard deviation of the pixel intensity. The luminance

and contrast for every stimulus are set to be identical. **(D)** Controlled intra-class similarity of the object stimulus ($n = 19{,}900$, two-sided rank-sum test, NS, $P > 0.767$). The intra-class similarity was measured as the image correlation between the images in each class. Error bars indicate the standard deviation ($n = 19{,}900$).

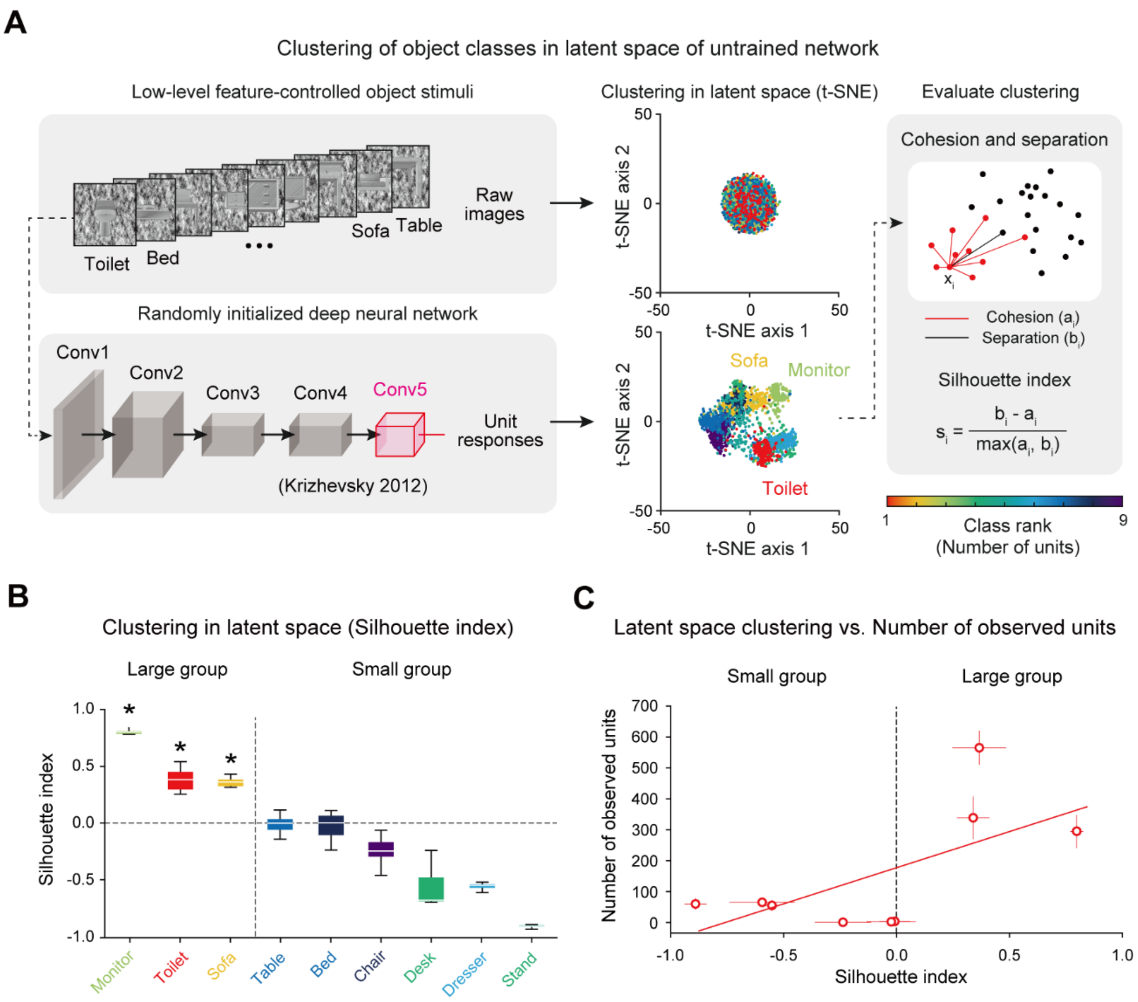

Supplementary Figure 2. Clustering of object classes in the latent space of untrained networks: **(A)** Overall process of evaluating the clustering of unit responses from untrained networks. (Left) Low-level feature-controlled object stimuli were used to obtain unit responses of a randomly initialized deep neural network. (Middle) Clustering of class representation in the latent space is shown by t-SNE analysis. (Right) Evaluation of clustering by silhouette index. **(B)** Silhouette index calculated from clustered unit responses across object classes. Note that object classes in the large group show significantly higher silhouette index (n = 20, one-sided signed rank-sum test, Monitor, $*P < 10^{-4}$; Toilet, $*P < 10^{-4}$; Sofa, $*P < 10^{-4}$). (C) Correlation between the silhouette index and the number of selective units (Pearson correlation coefficient, n = 20, r = 0.62, $P < 10^{-20}$). Box plots indicate the interquartile range (IQR between Q1 and Q3) of the dataset. White lines depict the median and whisker plots indicate the rest of the distribution (Q1 − 1.5*IQR, Q3 + 1.5*IQR). Error bars indicate the standard deviation of 20 random networks.

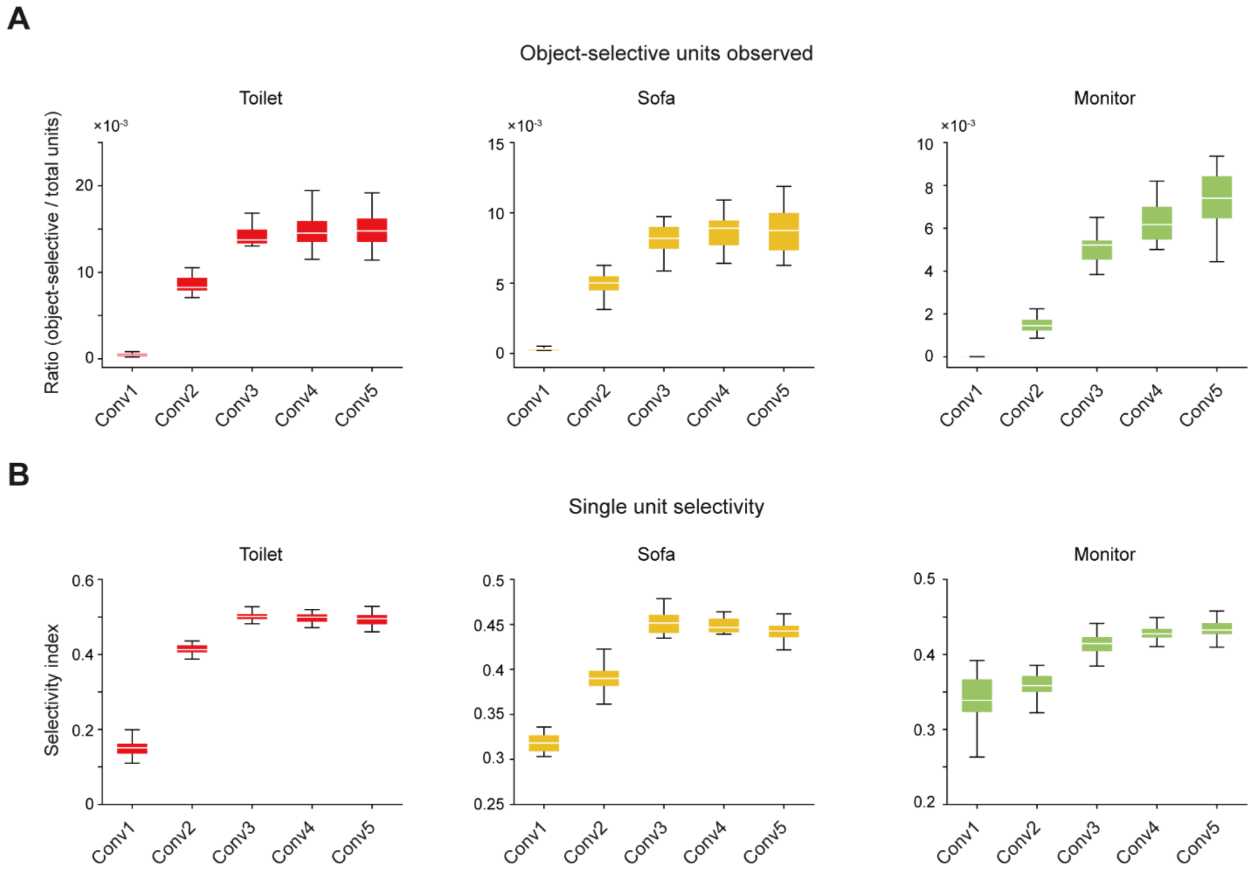

Supplementary Figure 3. Layer-dependent object selectivity across the hierarchal layer in untrained networks: **(A)** Number of selective units across convolutional layers in untrained networks (n = 20). **(B)** Object-selective index of single units (n = 20) across the convolutional layers. Box plots indicate the inter-quartile range (IQR between Q1 and Q3) of the dataset. White lines depict the median and whisker plots indicate the rest of the distribution (Q1 − 1.5*IQR, Q3 + 1.5*IQR).

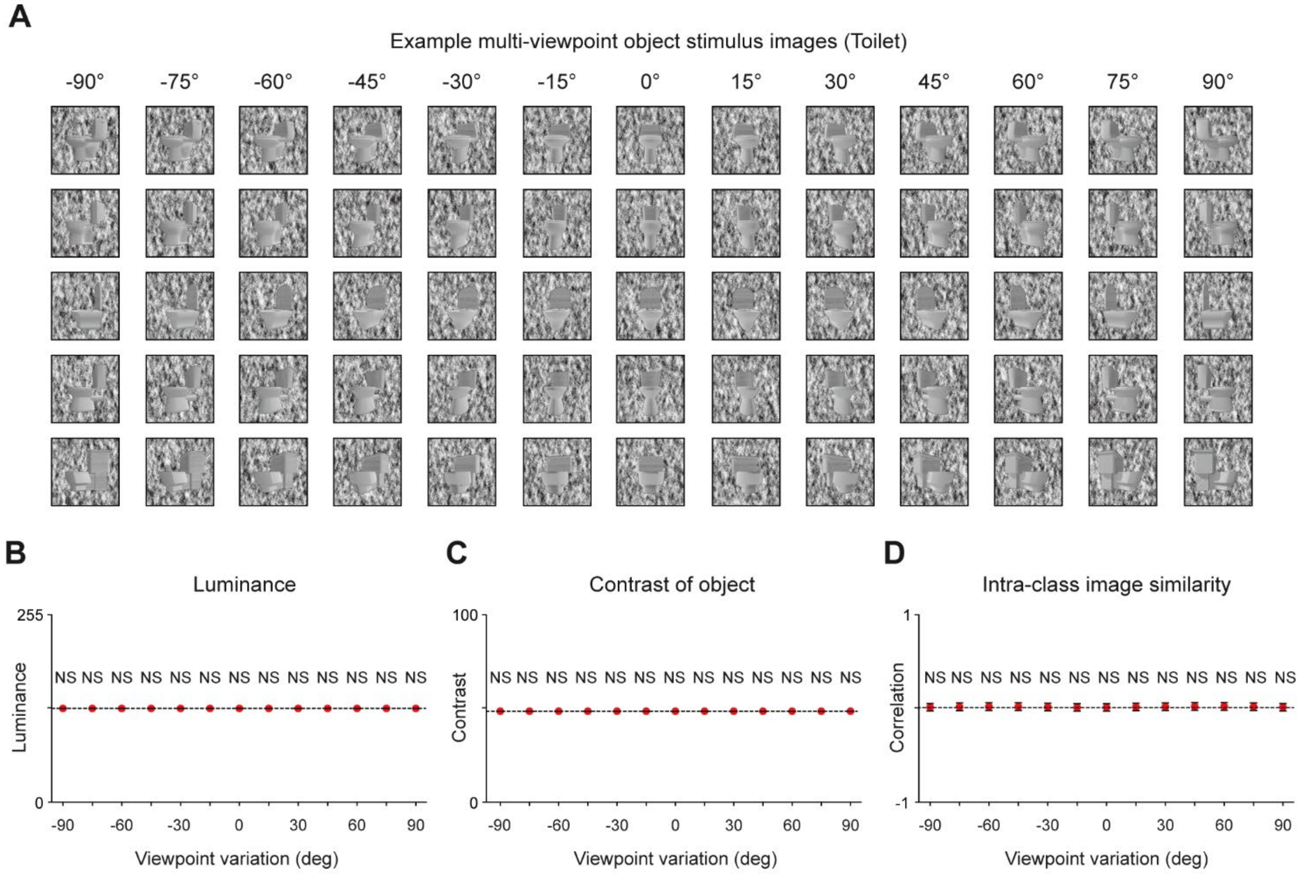

Supplementary Figure 4. Low-level feature-controlled object image stimulus with various viewpoints: **(A)** Example images of the viewpoint dataset. Using the viewpoint-controllable object stimulus renderer, a viewpoint dataset that contains 13 viewpoint classes from -180° to +180° was generated. **(B)** Controlled luminance of stimulus images (n = 200, two-sided rank-sum test, NS, P > 0.816). **(C)** Controlled contrast of stimulus images (n = 200, two-sided rank-sum test, NS, P > 0.485). The luminance and contrast for every stimulus are set to be identical. **(D)** Controlled intra-class similarity of the object stimulus (n = 19,900, two-sided rank-sum test, NS, P > 0.492). The intra-class similarity was measured as the image correlation between the images in each class. Error bars indicate the standard deviation (n = 19,900).

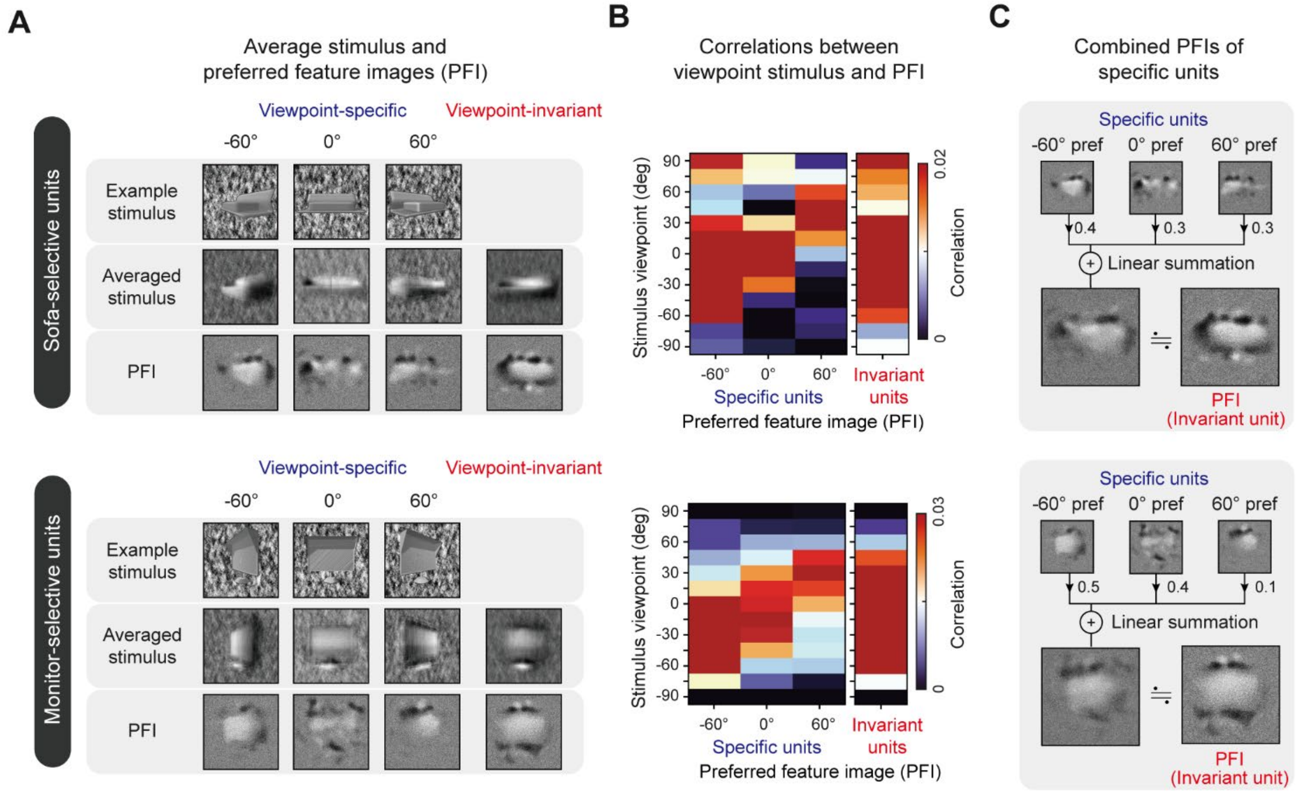

Supplementary Figure 5. Preferred feature image (PFI) analysis for specific and invariant units: **(A)** Example PFIs of invariant and specific units. An example stimulus and the average stimulus are also presented together for a visual comparison. **(B)** Correlation between the PFIs of each type of unit and stimulus with different angles. **(C)** Linear combination of PFIs of specific units showing the highest correlation with the PFIs of invariant units.

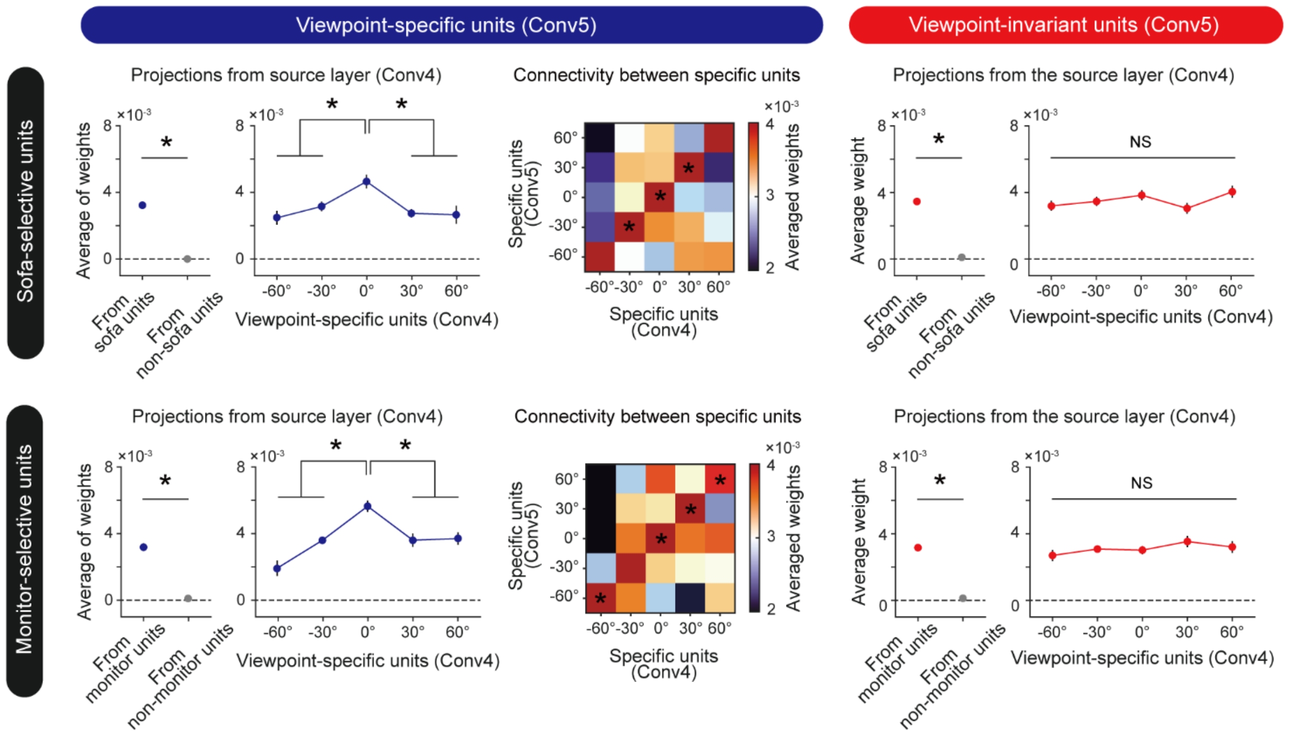

Supplementary Figure 6. Emergence of viewpoint invariance: In each panel, the leftmost graph demonstrates how strongly a target unit is connected to each type of unit in the source layer (n = 20, two-sided rank-sum test; Specific sofa unit, *P < 10^{-7}; Invariant sofa unit, *P < 10^{-7}; Specific monitor unit, *P < 10^{-7}; Invariant monitor unit, *P < 10^{-7}). The graph next to the leftmost graph indicates how strongly a unit in the target layer is connected to specific units with different angles in the source layer (n = 20, one-way ANOVA; Specific sofa unit, *P < 10^{-3}; Invariant sofa unit, NS, P = 0.132; Specific monitor unit, *P < 0.05; Invariant monitor unit, NS, P = 0.991). Error bars indicate the standard error of 20 random networks. The heatmap, located at the center between two panels, shows how strongly the specific units with various preferred angles are connected between the source and target layers (n = 20, one-way ANOVA; Specific sofa unit, *P < 0.001; Specific monitor unit, *P < 0.05).

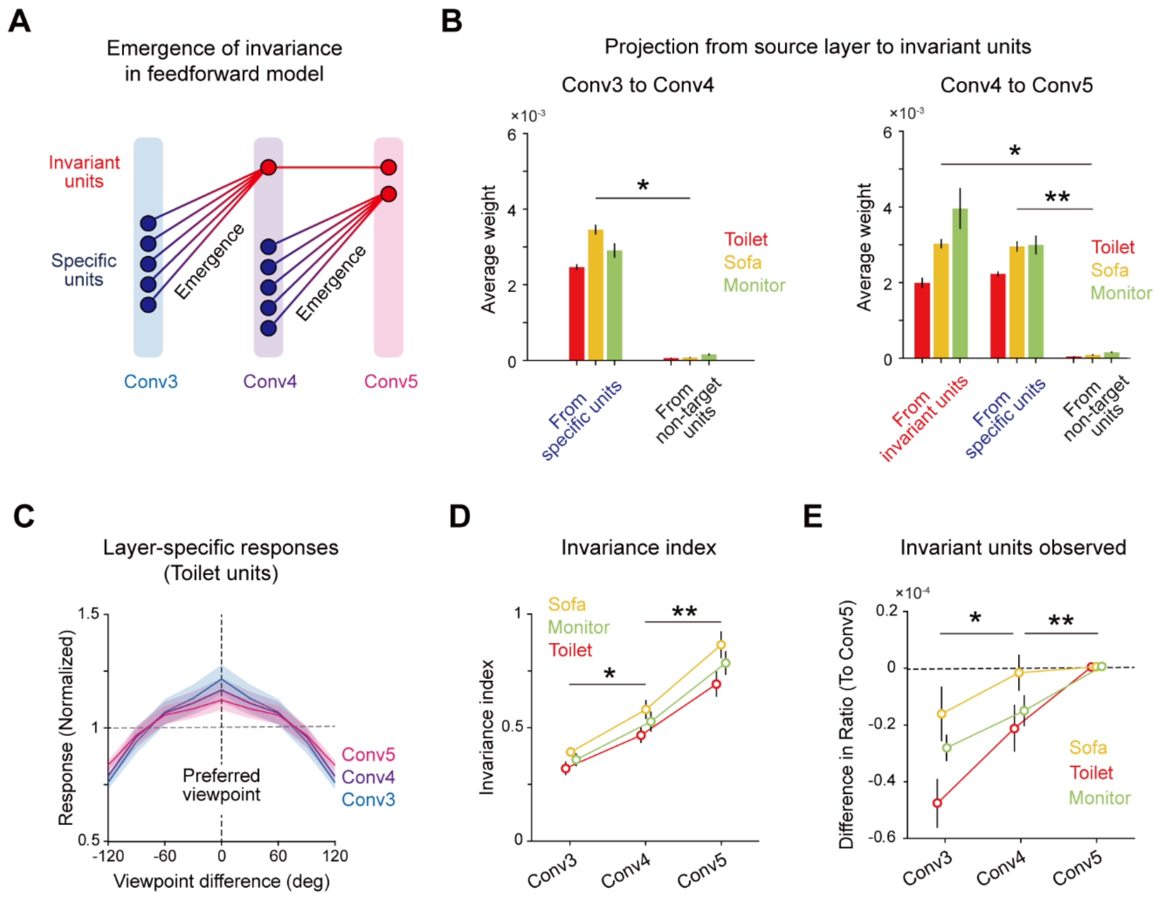

Supplementary Figure 7. Invariance across the hierarchal layer in untrained networks: **(A)** Schematic diagram of a feedforward model scenario on the emergence of invariant unit. **(B)** Feedforward projections from units in source layers to invariant units in target layers. Viewpoint invariant units in each layer are strongly connected to viewpoint specific units in previous layers, as predicted by the feedforward summation model (n = 20, two-sided rank-sum test, from invariant units, Conv4 to Conv5, Toilet: *$P < 10^{-7}$, Sofa: *$P < 10^{-7}$, Monitor: *$P < 10^{-7}$; from specific units, Conv3 to Conv4, Toilet: *$P < 10^{-7}$, Sofa: *$P < 10^{-7}$, Monitor: *$P < 10^{-6}$; Conv4 to Conv5, Toilet: **$P < 10^{-7}$, Sofa: **$P < 10^{-7}$, Monitor: **$P < 10^{-7}$). Bars and error bars correspondingly indicate the mean and the standard error of 20 random networks. **(C)** The average tuning curve of toilet-selective units in each convolutional layer. Note that the tuning curves become flatter as the layer deepens, demonstrating increased invariance of tuning. The shaded area indicates the standard error of all units in each layer. **(D)** Invariance index of the object units across convolutional layers. The invariance index of selective units increases higher up in the hierarchy (n = 20, two-sided rank-sum test; Toilet, *$P < 10^{-7}$, **$P < 10^{-7}$; Sofa, *$P < 10^{-7}$, **$P < 10^{-7}$; Monitor, *$P < 10^{-7}$, **$P < 10^{-7}$) **(E)** The ratio of invariant units across convolutional layers. Circles and error bars correspondingly indicate the mean and the standard error of 20 random networks in **(D)** and **(E)**.
